# P2X7 receptor mediates NLRP3-dependent IL-1β secretion and parasite proliferation in *Toxoplasma gondii*-infected human small intestinal epithelial cells

**DOI:** 10.1186/s13071-017-2573-y

**Published:** 2018-01-02

**Authors:** Juan-Hua Quan, Rui Huang, Zhuang Wang, Shuai Huang, In-Wook Choi, Yu Zhou, Young-Ha Lee, Jia-Qi Chu

**Affiliations:** 1 Department of Gastroenterology, Affiliated Hospital of Guangdong Medical University, Zhanjiang, Guangdong Province 524001 People’s Republic of China; 2 Stem Cell Research and Cellular Therapy Center, Affiliated Hospital of Guangdong Medical University, Zhanjiang, Guangdong Province 524001 People’s Republic of China; 3 Department of Infection Biology, Chungnam National University School of Medicine, Daejeon, 35015 Republic of Korea

**Keywords:** *Toxoplasma gondii*, Human small intestinal epithelial cells, P2X7 receptor, NLRP3 inflammasome, IL-1β, Proliferation

## Abstract

**Background:**

*Toxoplasma gondii* can invade and replicate in all nucleated cells in a wide range of host species, and infection induces IL-1β production. IL-1β plays central roles in the stimulation of the innate immune system and inflammation. However, little is known of the innate immune responses in human fetal small intestinal epithelial cells (FHs 74 Int cells) after *T. gondii* infection.

**Methods:**

FHs 74 Int cells were infected with the *T. gondii* GFP-RH strain. Then, IL-1β production and its mechanisms of action were evaluated using ELISA, MTT cell viability assays, Western blotting, immunofluorescence, quantitative real-time polymerase chain reaction (qRT-PCR), and gene-specific small interfering RNA (siRNA) transfection.

**Results:**

Infection of FHs 74 Int cells by *T. gondii* triggered significant time- and dose-dependent IL-1β production. Although *T. gondii* activated NLRP1, NLRP3, NLRC4 and AIM2 inflammasomes in FHs 74 Int cells, NLRP3 levels were consistently and significantly time-dependently increased, while the other inflammasomes were not. Transfection with siRNA targeting NLRP3, cleaved caspase-1 (Casp-1) or ASC significantly reduced *T. gondii*-induced IL-1β production, whereas *T. gondii* proliferation was markedly increased. *Toxoplasma gondii* infection activated P2X7 receptor (P2X7R) levels in FHs 74 Int cells in a time-dependent manner; however, transfection with siRNA targeting P2X7R significantly reduced *T. gondii*-induced IL-1β secretion and substantially increased *T. gondii* proliferation, which is mediated by decreased protein expression levels of NLRP3, cleaved Casp-1 and ASC. Collectively, NLRP3-dependent IL-1β secretion is mediated by P2X7R in small intestinal epithelial cells in response to *T. gondii* infection, thereby controlling parasite proliferation.

**Conclusions:**

This study revealed that the P2X7R/NLRP3 pathway plays important roles in IL-1β secretion and inhibition of *T. gondii* proliferation in small intestinal epithelial cells. These results not only contribute to our understanding of the mucosal immune mechanisms of *T. gondii* infection but also offer new insight into the identification of innate resistance in the gut epithelium.

**Electronic supplementary material:**

The online version of this article (10.1186/s13071-017-2573-y) contains supplementary material, which is available to authorized users.

## Background

*Toxoplasma gondii* is an obligate intracellular protozoan parasite that can invade and replicate in all nucleated cells. It is prevalent in humans and animals worldwide, and one-third of the world’s population is reportedly infected with *T. gondii* [1]. Human exposure to *T. gondii* typically results from the ingestion of cysts in contaminated food or water. Oocysts containing highly infectious sporozoites are shed by infected felids, which is the definitive host of *T. gondii*. A single infected cat can shed millions of oocysts into the environment. These cysts can be taken up by intermediate hosts, which include virtually any warm-blooded animal, including humans. In these infected intermediate hosts, *T. gondii* establishes a chronic infection in the form of bradyzoite-containing tissue cysts [2]. Humans also can be infected by consuming undercooked meat from intermediate hosts that harbor tissue cysts, but the parasite then breaches the intestinal epithelial barrier and spreads from *lamina propria* to a variety of other organs in the body [3]. Intestinal epithelial cells can sense and respond to microbial stimuli to reinforce their barrier function and to participate in the coordination of appropriate immune responses [4]. *Toxoplasma gondii* invades the intestinal epithelium, where it provokes appropriate immune responses that depend on local and systemic conditions [1, 5, 6]. However, the exact roles of the small intestinal epithelium in the activation of innate immunity against *T. gondii* infection remain poorly understood.

The innate immune system plays key roles in sensing pathogens and triggering biological mechanisms to control infection and eliminate pathogens [7, 8]. It is activated when pattern-recognition sensor proteins, such as Toll-like receptors (TLRs) or nucleotide-binding and oligomerization domain (NOD)-like receptors (NLRs), detect the presence of pathogens, their products, or danger signals [7–9]. NLRs are a large group of cytosolic receptors that are important modulators of inflammation through their regulation of pro-inflammatory cytokines IL-1β and IL-18 and due to their role in the pro-inflammatory form of cell death [10–12]. Once a ligand binds NLRs, oligomerization occurs with procaspase-1 and the adaptor molecule apoptosis-associated speck-like protein containing carboxy-terminal caspase activation and recruitment domain (ASC) to form a multimeric protein complex termed an inflammasome. Activation of inflammasomes triggers self-cleavage and activation of pro-Casp-1 to an active protease, which then cleaves cytosolic pro-IL-1β and pro-IL-18 into their active forms [11, 12]. There are some reports regarding the activation of inflammasomes in *T. gondii*-infected macrophages or monocytes [13–18]. However, the roles of inflammasomes, especially NLRP3, and the associated regulatory pathways in *T. gondii*-infected intestinal epithelial cells have not been studied.

The IL-1 cytokine family consists of 11 members with different roles in inflammation. IL-1β is recognized as one of the earliest and most potent pro-inflammatory agents synthesized and released in response to infectious agents and injuries [19, 20]. Under normal conditions, IL-1β is not constitutively expressed but rather is induced by microbial products or endogenous signals. Blood monocytes, tissue macrophages and dendritic cells are the primary sources of IL-1β production [11, 12, 18–20].

The purinergic P2X7 receptor (P2X7R) is a bi-functional adenosine triphosphate (ATP)-gated plasma membrane ion channel that contributes to the control of many physiological functions, including cell death, killing infectious organisms, and regulating inflammatory processes [21–23]. P2X7R is widely distributed in human tissues, with the highest expression in cells of the immune and inflammatory systems. Activation of P2X7R triggers inflammasome formation, culminating in mature IL-1β release, and participates in pro-inflammatory events [21–23]. Some researchers have studied the expression of P2X7R in immune cells or mice after *T. gondii* infection [24–27].

Although many reports have described IL-1β production by macrophages and dendritic cells after *T. gondii* infection [13, 17, 24, 25], IL-1β production and its regulatory pathways in intestinal epithelial cells during *T. gondii* infection have not been presented. Thus, we investigated IL-1β production and its roles in human fetal small intestinal epithelial cells (FHs 74 Int cells) after *T. gondii* infection using ELISA, MTT cell viability assays, Western blotting, immunofluorescence, quantitative real-time polymerase chain reaction (qRT-PCR), and gene-specific small interfering RNA (siRNA) transfection.

## Methods

### Maintenance of *T. gondii*

Tachyzoites of the *T. gondii* RH strain, which expresses transgenic green fluorescent protein (GFP-RH), were maintained as described previously [28] with minor modifications. Briefly, human retinal pigment epithelial cells (ARPE-19 cells) (ATCC, Manassas, VA, USA) were cultured in a 1:1 (*v*/v) mixture of DMEM/F12 supplemented with 10% (v/v) heat-inactivated fetal bovine serum (FBS) and an antibiotic-antimycotic solution (all from Gibco, Grand Island, NY, USA). ARPE-19 cells were infected with *T. gondii* at a multiplicity of infection (MOI) of 5 and incubated at 37 °C under 5% (v/v) CO_2_ for 2–3 days. After spontaneous host cell rupture, parasites and cellular debris were pelleted by centrifugation and washed in cold PBS. The final pellet was resuspended and passed through a 26-gauge needle fitted with a 5.0 μm pore-sized filter (Millipore, Billerica, MA, USA).

### Culture of FHs 74 Int cells

A non-transformed human fetal small intestinal epithelial cell line (FHs 74 Int cells) was purchased from ATCC and cultured in DMEM with 10% (*v*/v) FBS, an antibiotic-antimycotic solution, and 30 ng/ml human epidermal growth factor (all from Gibco) at 37 °C in a humidified atmosphere at 5% (v/v) CO_2_. The medium was changed every 2–3 days.

### IL-1β assay

FHs 74 Int cells were mock-infected (negative control) or infected with the *T. gondii* GFP-RH strain at different MOIs for 8 h or at an MOI of 10 for various time periods. Triplicate supernatants from mock-infected or *T. gondii*-infected cells were collected, and IL-1β levels were measured using a commercial ELISA kit according to the manufacturer’s instructions (eBioscience, San Diego, CA, USA). Cytokine concentrations in the samples were calculated using standard curves based on the known quantities of recombinant cytokines.

### MTT cell viability assay

A 3-(4,5-dimethylthiazol-2-yl)-2,5-diphenyltetrazolium bromide (MTT) assay was performed according to the manufacturer’s instructions. FHs 74 Int cells were plated in triplicate in 96-well plates at a density of 2500 cells/well and infected at MOIs of 1, 2, 5, 10 or 20. After 8 h, 5 mg/ml MTT (Sigma-Aldrich, St. Louis, MO, USA) in PBS was added to each well at a final concentration of 0.5 mg/ml, and the plates were incubated at 37 °C for 4 h. Subsequently, formazan was dissolved in 100 μl of crystal dissolving buffer (0.04 N HCl in absolute isopropanol) and added to each well. The optical density at 490 nm was then measured using a Multiscan GO microplate reader (Thermo Fisher Scientific, Waltham, MA, USA).

### Western blotting

Control FHs 74 Int cells or siRNA-transfected FHs 74 Int cells were mock-infected or infected with the *T. gondii* GFP-RH strain as indicated. After the cells were washed with ice-cold PBS, proteins were extracted with RIPA buffer (Thermo Fisher Scientific, Grand Island, NY, USA). Protein concentrations were determined using the Bradford assay (Bio-Rad, Hercules, CA, USA). Total protein (30 μg) was resolved on 10–12% SDS-PAGE gels and then transferred to PVDF membranes (Merck Millipore, Billerica, MA, USA). The membranes were blocked with 5% nonfat skim milk in TBS containing 0.1% Tween 20 (TBST) for 1 h and incubated with primary antibodies against IL-1β, NLRP1, NLRP3, NLRC4, AIM2, cleaved Casp-1 (Asp297), Casp-1, ASC, P2X7 receptor (P2X7R) (all from Cell Signaling Technology, Danvers, MA, USA), or α-Tubulin (Santa Cruz Biotechnology, Santa Cruz, CA, USA) overnight at 4 °C. Subsequently, the membranes were incubated with HRP-conjugated secondary antibody (Santa Cruz Biotechnology) for 2 h at room temperature. Blots were developed using a commercially available enhanced chemiluminescence (ECL) detection kit (GE Healthcare, Little Chalfont, UK). These experiments were repeated at least three times with similar results. Quantification of band intensity was performed using ImageJ software (NIH, Bethesda, Maryland, USA). The results were normalized to α-Tubulin protein levels and are expressed as fold changes over the mock-infection control group.

### Quantitative real-time polymerase chain reaction (qRT-PCR)

FHs 74 Int cells were mock-infected or infected with the GFP-RH strain of *T. gondii* at an MOI of 10 for 4 or 8 h. Total cellular RNA was extracted using TRIzol Reagent (Invitrogen Life Technologies, Carlsbad, CA, USA), and 3 μg RNA was reverse-transcribed in a final volume of 20 μl using Superscript II reverse transcriptase (Invitrogen Life Technologies) as described by the manufacturer. qRT-PCR was performed using SYBR Premix Ex Taq II reagent (Takara Bio Inc., Dalian, China) as described previously [3]. Each reaction included 1 μl of cDNA (100 ng/μl), 10 μl of SYBR Premix Ex Taq II (2X), 0.8 μl of forward and reverse primers (10 μM), 0.4 μl of ROX reference Dye II and 7 μl of DNase-/RNase-free PCR water to a final volume of 20 μl. The primers used in this study are summarized in Additional file 1: Table S1. All reactions were performed with an ABI 7500 Fast Real-Time PCR system (Applied Biosystems, Carlsbad, CA, USA) under the following conditions: 95 °C for 30 s, followed by 40 cycles of 95 °C for 5 s and 60 °C for 34 s. Relative gene expression levels were quantified based on the cycle threshold (Ct) values and were normalized to the reference gene hypoxanthine phosphoribosyltransferase 1 (HPRT1). Each sample was measured in triplicate, and the gene expression levels were calculated using the 2^–ΔΔCt^ method.

### siRNA transfection

Scrambled negative-control siRNA and siRNAs specific for NLRP3, Casp-1, ASC and P2X7R were purchased from Santa Cruz Biotechnology. FHs 74 Int cells were transfected with the siRNAs using Lipofectamine RNAiMAX (Invitrogen Life Technologies) according to the manufacturer’s instructions. Briefly, cells were seeded in 6-well plates and grown for 24 h (70% confluence) prior to transfection. Nine microliters of Lipofectamine RNAiMAX Reagent was diluted with 150 μl of Opti-MEM and 3 μl of siRNA in 150 μl of Opti-MEMR from a 10 μM stock solution. Diluted siRNA was added to diluted Lipofectamine RNAiMAX Reagent and incubated at RT for 5 min. Finally, the siRNA-lipid complex was added to the cells, and the cells were incubated for 72 h at 37 °C in a humidified atmosphere at 5% (*v*/v) CO_2_. The knockdown efficiency was determined by Western blotting. All experiments were repeated independently three times.

### Immunofluorescence assay

To measure *T. gondii* infection and replication rates, monolayers of siRNA-transfected FHs 74 Int cells on glass coverslips were infected with parasites at an MOI of 10 for 2 or 8 h. The cells were then washed several times with PBS, fixed with 4% (v/v) paraformaldehyde, and permeabilized with 0.1% (v/v) Triton X-100 in PBS (PBST) for 10 min. The coverslips were then washed with PBST and stained with Texas Red-X phalloidin (Life Technologies) to label F-actin. Finally, the coverslips were washed and mounted onto microscopy slides using a mounting medium containing DAPI (to detect nuclei) (Vector Laboratories, Burlingame, CA, USA), and the cells were imaged using fluorescence microscopy. All experiments were performed on triplicate samples.

### Statistical analysis

All results are presented as the means ± standard deviations (SDs) of at least three independent experiments, unless otherwise indicated. Statistical significance was determined using an unpaired Student’s *t*-test or one-way ANOVA. A *P*-value < 0.05 was considered significant.

## Results

### *Toxoplasma gondii* induces IL-1β secretion in small intestinal epithelial cells

**Fig. 1:**
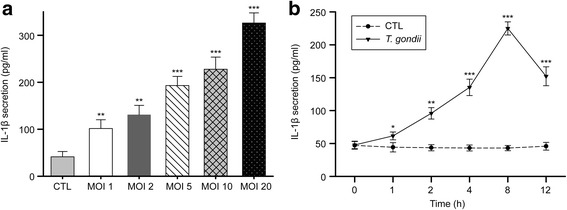
*T. gondii*-induced IL-1β secretion in FHs 74 Int cells. **a** Cells were infected with the *T. gondii* GFP-RH strain at the indicated MOIs for 8 h, and mature IL-1β levels in the cell culture supernatants were assessed by ELISA. **b** Cells were infected with *T. gondii* at an MOI of 10 for different time periods, and mature IL-1β levels in the cell culture supernatants were assessed by ELISA. *P < 0.05, **P < 0.01, ***P < 0.001 compared with the mock-infection control. All data are representative of three independent experiments

To evaluate the capacity of small intestinal epithelial cells to produce IL-1β in response to *T. gondii*, FHs 74 Int cells were infected with live *T. gondii* at different MOIs for 8 h, and IL-1β levels in the cell culture supernatants were measured by ELISA. As shown in Fig. 1a, *T. gondii* significantly enhanced IL-1β secretion in an MOI-dependent manner (MOI 1: *t*_(4)_ = 4.78, *P* = 0.009; MOI 2: *t*_(4)_ = 6.756, *P* = 0.0025; MOI 5: *t*_(4)_ = 11.72, *P* = 0.0003; MOI 10: *t*_(4)_ = 11.52, *P* = 0.0003; MOI 20: *t*_(4)_ = 20.36, *P* < 0.0001). However, the cell viability of FHs 74 Int cells infected with *T. gondii* at an MOI of 20 was slightly reduced compared with that of uninfected control cells (Additional file 2: Figure S1). Thus, we chose an MOI of 10 to establish the *T. gondii* infection model of FHs 74 Int cells. We then assessed the time course of IL-1β secretion from FHs 74 Int cells after *T. gondii* infection. We found that IL-1β secretion was significantly increased 1–12 h post-infection compared with that of the mock-infection control; IL-1β levels peaked at 8 h and then gradually decreased (1 h: *t*_(4)_ = 2.887, *P* = 0.045; 2 h: *t*_(4)_ = 8.039, *P* = 0.0013; 4 h: *t*_(4)_ = 11.06, *P* = 0.0004; 8 h: *t*_(4)_ = 26.69, *P* < 0.0001; 12 h: *t*_(4)_ = 11.69, *P* = 0.0003) (Fig. 1b). Similarly, pro-IL-1β protein levels significantly increased after *T. gondii* infection in a time-dependent manner, peaking at 8 h post-infection (2 h: *t*_(4)_ = 4.067, *P* = 0.015; 4 h: *t*_(4)_ = 10.67, *P* = 0.0004; 8 h: *t*_(4)_ = 13.39, *P* = 0.0002; 12 h: *t*_(4)_ = 8.333, *P* = 0.0011) (Additional file 2: Figure S2). Thus, *T. gondii* infection of FHs 74 Int cells induced significant IL-1β production and secretion in time- and dose-dependent manners.

### The NLRP3 inflammasome was consistently and significantly increased with time in *T. gondii*-infected small intestinal epithelial cells

**Fig. 2:**
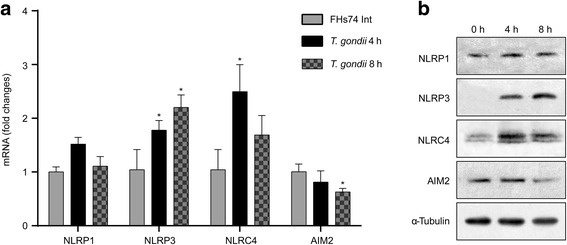
*Toxoplasma gondii* significantly increased NLRP3 expression levels in a time-dependent manner. FHs Int 74 cells were mock-infected or infected with the *T. gondii* GFP-RH strain at an MOI of 10. Cells were harvested 4 or 8 h post-infection. **a** NLRP1, NLRP3, NLRC4 and AIM2 expression levels were determined by qRT-PCR. HPRT1 served as an internal control. *P < 0.05 compared with the mock-infection control. **b** NLRP1, NLRP3, NLRC4 and AIM2 protein levels in cell extracts were determined by Western blotting. All data are representative of three independent experiments

We next explored the involvement of specific inflammasomes in IL-1β secretion after *T. gondii* infection. According to previous reports, NLRP1, NLRP3, NLRC4 and AIM2 inflammasomes are activated in *T. gondii*-infected DC or macrophages [13, 14, 17, 18]. Thus, we selected these 4 inflammasomes (NLRP1, NLRP3, NLRC4 and AIM2) and investigated the expression of each inflammasome in FHs 74 Int cells after *T. gondii* infection using qRT-PCR and Western blotting. As shown in Fig. 2, both the mRNA and proteins levels of NLRP3 inflammasomes were significantly increased in a time-dependent manner in *T. gondii*-infected FHs 74 Int cells. However, NLPR1 expression was not significantly changed after infection. NLRC4 mRNA levels were significantly upregulated 4 h after *T. gondii* infection but decreased slightly after 8 h (4 h: *t*_(4)_ = 3.998, *P* = 0.016). In addition, AIM2 mRNA levels were significantly decreased at 8 h post-infection (*t*_(4)_ = 4.213, *P* = 0.014). According to the mRNA and protein expression levels of 4 inflammasomes in *T. gondii*-infected FHs 74 Int cells, NLRP3 expression was consistently and significantly increased with time, while the others were not (4 h: *t*_(4)_ = 3.047, *P* = 0.038; 8 h: *t*_(4)_ = 4.509, *P* = 0.011). Therefore, we focused on the NLRP3 inflammasome. We next evaluated whether NLRP3 inflammasome is involved in *T. gondii-*induced IL-1β production and whether they exert protective effects in FHs 74 Int cells. In future studies, we plan to evaluate the roles of other inflammasomes.

### *Toxoplasma gondii* induces IL-1β secretion from small intestinal epithelial cells via the NLRP3 inflammasome

**Fig. 3:**
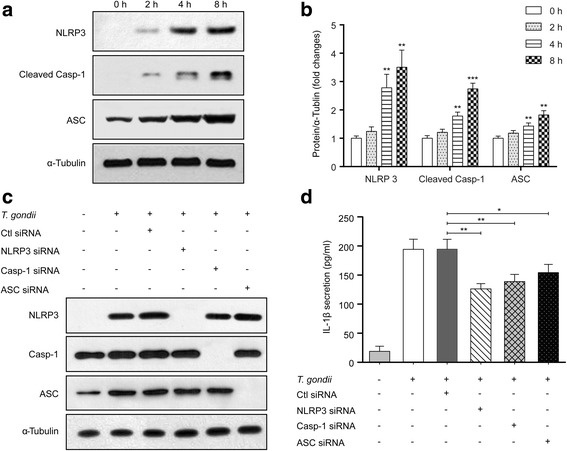
*Toxoplasma gondii* induces IL-1β production in FHs Int 74 cells via activation of the NLRP3 inflammasome. FHs 74 Int cells were infected with *T. gondii* at an MOI of 10 for 0, 2, 4 or 8 h. The cells were then collected and analyzed for NLRP3, cleaved Casp-1 and ASC protein levels by Western blotting. **a** Representative blots depicting NLRP3, cleaved Casp-1 and ASC protein levels in mock-infected or *T. gondii*-infected FHs 74 Int cells (α-Tubulin served as an internal control for protein loading). **b** Bar plot depicting the NLRP3/α-Tubulin, cleaved Casp-1/α-Tubulin and ASC/α-Tubulin ratios as determined by densitometric analysis of Western blotting and expressed as fold change compared with the mock-infection control. For all panels, data are presented as the mean ± SD. *P < 0.05, **P < 0.01, ***P < 0.001 compared with mock-infection control. FHs 74 Int cells were transfected with NLRP3, Casp-1, ASC or control siRNAs and then infected with *T. gondii* at an MOI of 10 for 8 h. **c** Cell extracts were subjected to Western blotting to detect NLRP3, Casp-1 and ASC protein levels. **d** The cell culture supernatants were collected and assayed for IL-1β levels by ELISA. *P < 0.05, **P < 0.01 compared with control siRNA-transfected *T. gondii*-infected cells. All data shown are representative of three independent experiments

The NLRP3 inflammasome complex consists of the NLRP3 sensor, the adaptor bipartite protein known as ASC, and pro-caspase-1 [6, 7]. To better understand the kinetics of the NLRP3 inflammasome activation, we investigated each component of NLRP3 inflammasome at different time-points after infection with *T. gondii*. As shown in Fig. 3a, b, *T. gondii* significantly upregulated NLRP3 (4 h: *t*_(4)_ = 6.411, *P* = 0.003; 8 h: *t*_(4)_ = 7.124, *P* = 0.002) and cleaved Casp-1 (4 h: *t*_(4)_ = 8.112, *P* = 0.0013; 8 h: *t*_(4)_ = 13.71, *P* = 0.0002) and ASC (4 h: *t*_(4)_ = 5.494, *P* = 0.005; 8 h: *t*_(4)_ = 8.471, *P* = 0.0011) protein levels in a time-dependent manner (from 2 to 8 h). To determine whether *T. gondii* mediates IL-1β release via the NLRP3 inflammasome, we transfected cells with siRNAs specific to NLRP3, Casp-1 or ASC for 72 h and then infected the cells with *T. gondii* GFP-RH at an MOI of 10 for 8 h. Cell extracts were subjected to Western blotting to detect the knockdown efficiencies of NLRP3, Casp-1 and ASC. As shown in Fig. 3c, siRNA transfection markedly inhibited NLRP3, Casp-1 and ASC protein levels. ELISA of the supernatants confirmed that NLRP3 siRNA significantly suppressed *T. gondii*-induced IL-1β secretion compared with secretion in *T. gondii*-infected control siRNA cells (*t*_(4)_ = 6.219, *P* = 0.003). Similarly, Casp-1 (*t*_(4)_ = 4.649, *P* = 0.0097) and ASC (*t*_(4)_ = 3.193, *P* = 0.033) siRNAs also significantly reduced *T. gondii*-induced IL-1β secretion, although IL-1β levels were not drastically attenuated (Fig. 3d). Taken together, these results suggest that the NLRP3 inflammasome is important for *T. gondii*-induced IL-1β production and secretion in FHs 74 Int cells and that each component of the NLRP3 inflammasome is involved in IL-1β production.

### The NLRP3 inflammasome inhibits *T. gondii* proliferation in intestinal epithelial cells

**Fig. 4:**
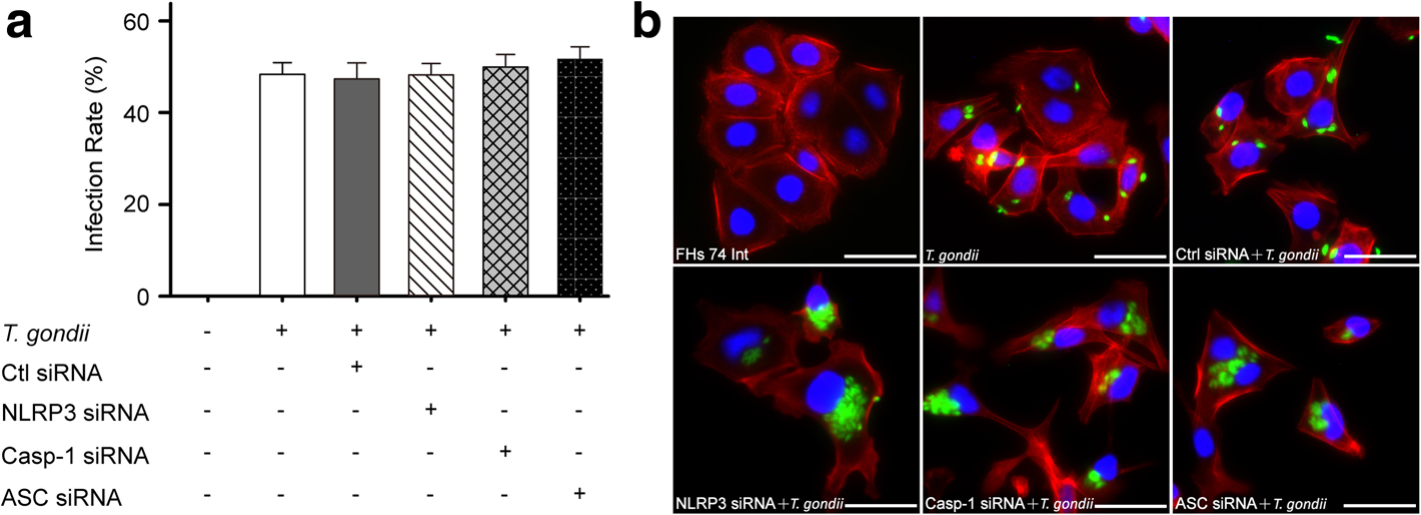
*T. gondii*-induced NLRP3 inflammasome activation inhibits intracellular parasite proliferation. **a** FHs 74 Int cells were transfected with NLRP3, Casp-1, ASC or control siRNAs and then infected with *T. gondii* at an MOI of 10 for 2 h. Cells were fixed and stained with Texas Red-X phalloidin to label F-actin (red), and the nuclei were stained with DAPI (blue). The number of *T. gondii*-infected cells and the total number of cells were counted, and the *T. gondii* infection rates were calculated. **b** siRNA-transfected FHs 74 Int cells were infected with *T. gondii* at an MOI of 10 for 8 h. Cells were fixed and stained with Texas Red-X phalloidin and DAPI. Intracellular parasites were revealed by fluorescence microscopy. All data shown are representative of three independent experiments. *Scale-bars*: 20 μm

To evaluate the roles of the NLRP3 inflammasome in protection against infection, we measured parasite infection and proliferation rates in transfected FHs 74 Int cells. We transfected FHs 74 Int cells with siRNAs targeting NLRP3, Casp-1 or ASC and then infected the cells with *T. gondii* GFP-RH at an MOI of 10 for 2 h. The cells were stained with Texas Red-X phalloidin (red) and DAPI (blue) to identify F-actin and nuclei, respectively. The number of *T. gondii*-infected cells and the total number of cells were counted under a fluorescence microscope, and the *T. gondii* infection rate was calculated. There was no significant difference in *T. gondii* infection rate in cells transfected with NLRP3, Casp-1 or ASC siRNA compared with those in control siRNA-transfected cells (Fig. 4a). To evaluate the effect of NLRP3 inflammasome activation on *T. gondii* proliferation, siRNA-transfected FHs 74 Int cells were infected with *T. gondii* at an MOI of 10 for 8 h, and intracellular parasite numbers were evaluated. Transfection with NLRP3, Casp-1 or ASC siRNA significantly increased intracellular parasite numbers compared with control siRNA-transfected *T. gondii*-infected by fluorescence microscopy (NLRP3: *t*_(4)_ = 20.92, *P* < 0.0001; Casp-1: *t*_(4)_ = 7.525, *P* = 0.0017; ASC: *t*_(4)_ = 4.504, *P* = 0.011) (Fig. 4b). Taken together, these data strongly suggest that the NLRP3 inflammasome inhibits parasite proliferation in *T. gondii*-infected FHs 74 Int cells.

### P2X7R is required for *T. gondii*-induced NLRP3 inflammasome activation

**Fig. 5:**
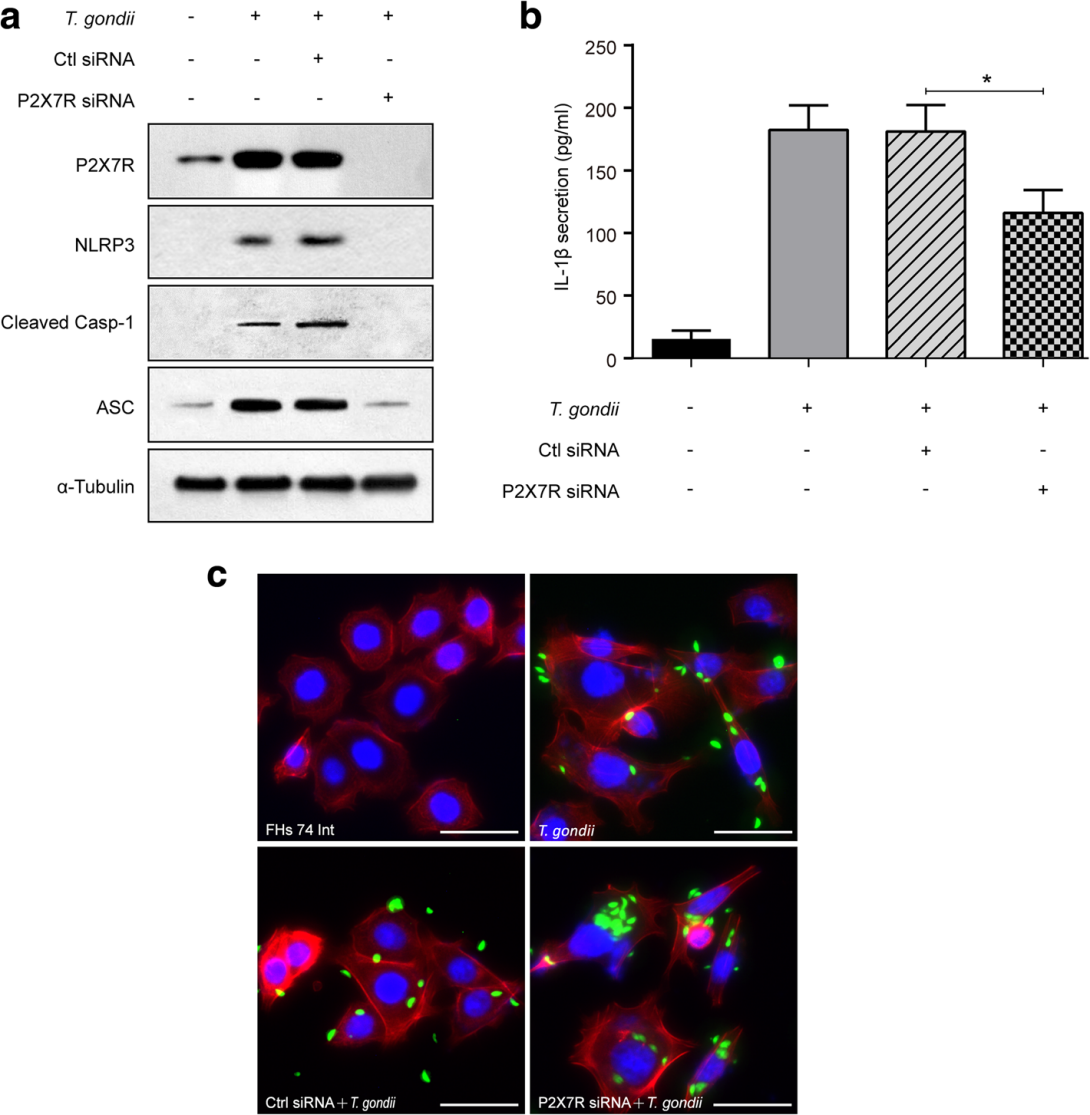
The P2X7R pathway is required for *T. gondii*-induced NLRP3 activation. P2X7R or control siRNA was transfected into FHs 74 Int cells that were then infected with the *T. gondii* GFP-RH strain at an MOI of 10 for 8 h. **a** Cell extracts were subjected to Western blotting to detect P2X7R, NLRP3, cleaved Casp-1 and ASC expression levels. **b** IL-1β levels in the cell culture supernatants were measured by ELISA. *P < 0.05 compared with control siRNA-transfected *T. gondii*-infected cells. **c** Cells were fixed and stained with Texas Red-X phalloidin to label F-actin (red), and the nuclei were stained with DAPI (blue). Intracellular parasites were revealed by fluorescence microscopy. All data shown are representative of three independent experiments. *Scale-bars*: 20 μm

We next explored the activation mechanism of the NLRP3 inflammasome in response to *T. gondii*. A recent study reported that P2X7R, a powerful activator of the NLRP3 inflammasome, is responsible for both NLRP3 recruitment and activation [29]. To determine whether *T. gondii*-induced P2X7R is involved in NLRP3 inflammasome activation, we transfected cells with P2X7R siRNA prior to *T. gondii* infection. Western blotting results showed that transfection with P2X7R siRNA significantly reduced *T. gondii*-induced NLRP3, cleaved Casp-1 and ASC protein levels (Fig. 5a). Similar results were also observed for IL-1β production using ELISA; P2X7R siRNA significantly reduced IL-1β secretion but did not entirely eliminate *T. gondii*-induced IL-1β secretion (*t*_(4)_ = 4.07, *P* = 0.0152) (Fig. 5b). To evaluate the effect of P2X7R on intracellular parasite proliferation, P2X7R siRNA-transfected FHs 74 Int cells were infected with *T. gondii*, and the intracellular parasite load was examined by fluorescence microscopy. Interestingly, transfection with P2X7R siRNA significantly increased the intracellular parasite burden compared with control siRNA-transfected *T. gondii*-infected cells (*t*_(4)_ = 4.421, *P* = 0.0115) (Fig. 5c). Thus, activation of the NLRP3 inflammasome requires the *T. gondii*-induced P2X7R pathway and inhibits parasite proliferation in FHs 74 Int cells.

## Discussion

**Fig. 6:**
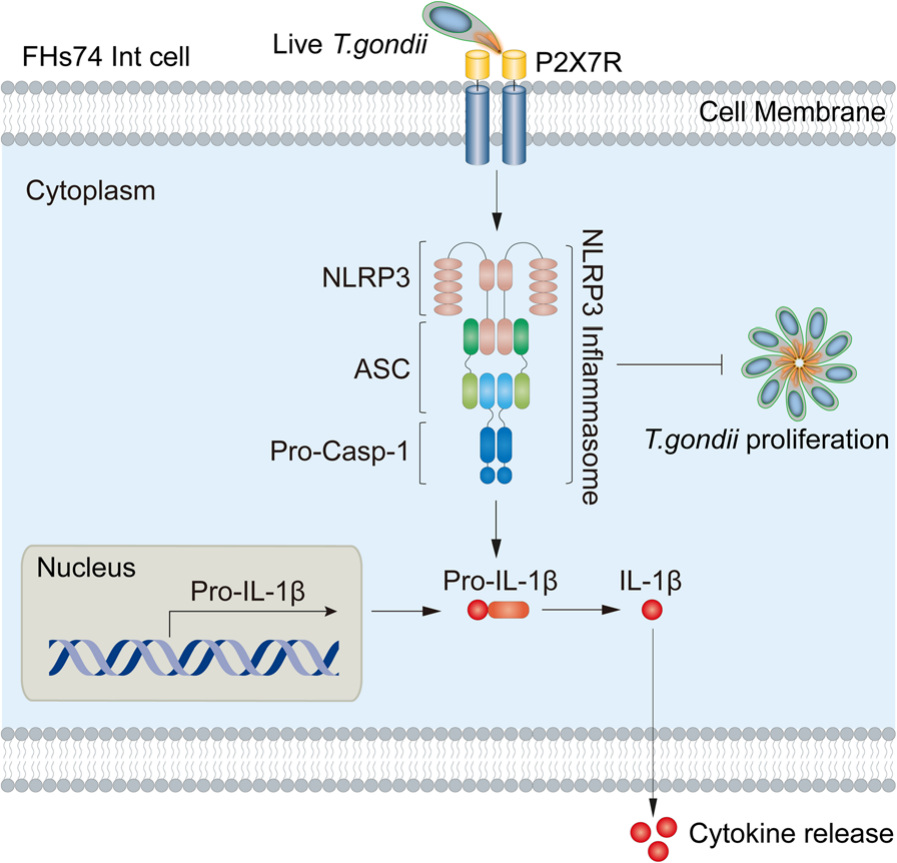
The proposed pathway of NLRP3 inflammasome activation in FHs 74 Int cells infected with *T. gondii*. P2X7R mediates activation of the NLRP3 inflammasome by forming a complex with the adaptor protein ASC and pro-Casp-1. Activation of the inflammasome induces IL-1β maturation and secretion. In addition, activation of the NLRP3 inflammasome inhibits *T. gondii* proliferation

*Toxoplasma gondii* can invade and replicate in the epithelial lining of the gastrointestinal tract and induce an immune response. IL-1β is a crucial factor of host defense in response to infections and injuries. Many studies have reported that *T. gondii* infection produces a significant amount of IL-1β in DCs, macrophages or monocytes but not in the intestinal epithelial lining [13–17, 25, 27]. Our data indicate that IL-1β production requires activation of the NLRP3 inflammasome in *T. gondii*-infected small intestinal epithelial cells, which is subsequently regulated by P2X7R and inhibits *T. gondii* proliferation (Fig. 6). To our knowledge, the present study for the first time suggests that P2X7R mediates NLRP3-dependent IL-1β secretion and parasite proliferation in *T. gondii*-infected human small intestinal epithelial cells.

IL-1β is recognized as one of the earliest and most potent pro-inflammatory agents, which is synthesized and released in response to infectious agents and injuries [19, 20, 23]. IL-1β was shown to be produced in murine bone marrow-derived macrophages, rat macrophages, and THP-1 macrophages after *T. gondii* infection through activation of inflammasomes [14, 15]. Interestingly, we show that *T. gondii* tachyzoites triggered significant IL-1β production by FHs 74 Int cells in time- and dose-dependent manners, indicating that the small intestinal epithelium has appropriate pattern-recognition sensor proteins to trigger inflammatory responses against the parasite. However*,* in vitro infection of mouse colonic epithelial CMT-93 cells by *T. gondii* PRU strain did not trigger inflammasome-associated IL-1β secretion [30]. These differences in IL-1β production may be the result of differences in cell types, host cell microenvironments, or the virulence of the *T. gondii* strain chosen.

Inflammasomes restrict microbial replication and trigger an inflammatory form of cell death, thus accounting for the genesis of inflammatory processes [11, 12, 18]. *T. gondii* induces the activation of NLRP1 and NLRP3 in macrophages and DCs [13–16], and *T. gondii* infection also activates NLRC4, NLRP6, NLRP8, NLRP13, AIM2, and NAIP in THP-1 macrophages [17]. Similarly, NLRP1, NLPR3, NLRC4 and AIM2 are four types of inflammasomes that were activated in *T. gondii*-infected intestinal epithelial cells, which is consistent with THP-1 macrophages [17]. However, their activation patterns differed. Among these four types of inflammasomes, the NLRP3 inflammasome was consistently and time-dependently activated. Moreover, the NLRP3 inflammasome is closely associated with P2X7 receptors [29, 31]. In the present study, *T. gondii*-induced IL-1β production was significantly reduced in the NLRP3 inflammasome component in siRNA-transfected FHs 74 Int cells, indicating that NLRP3 is involved in IL-1β production in *T. gondii*-infected human small intestinal epithelial cells.

We explored the activation mechanism and protective roles of the NLRP3 inflammasome in response to *T. gondii*. Under pathophysiological conditions of the cells, ATP released from dying cells enhances P2X7R activation to upregulate the NLRP3 inflammasome, subsequently promoting IL-1β secretion [21, 23]. P2X7R mediates NLRP3 inflammasome activation, cytokine and chemokine release, T lymphocyte survival and differentiation, transcription factor activation, and cell death [29]. Here, we found that transfection with P2X7R siRNA significantly reduced *T. gondii*-induced NLRP3, cleaved Casp-1 and ASC protein levels and IL-1β production in FHs 74 Int cells, whereas parasite proliferation was increased in *T. gondii*-infected NLRP3 or P2X7R siRNA-transfected FHs 74 Int cells. These results indicate that P2X7R and NLRP3 inflammasomes are important in the regulation of IL-1β production and control the resistance to this parasite. These findings were consistent with previous studies reporting that P2X7R-dependent responses play critical roles in the host control of *T. gondii* [24, 25]. However, in the present study, although the expression levels of the NLRP3 inflammasome and P2X7R were blocked by siRNA in host cells, *T. gondii*-induced IL-1β production was not dramatically suppressed. Regarding this phenomenon, increases in iNOS expression, NO production, and ROS generation may participate in the activation of the NLRP3 inflammasome and the subsequent regulation of IL-1β production [32]. In addition, a previous study shown that inflammatory caspases (caspases 1, 4, 5 and 11) are activated in response to microbial infection and danger signals. This process is followed by cleavage of gasdermin D (GSDMD) and the generation of an N-terminal cleavage product (GSDMD-NT) that triggers IL-1β inflammatory cytokine release [33]. A previous study and our results indicate that P2X7R-dependent NLRP3 inflammasome activation is required but not sufficient for *T. gndii*-induced IL-1β secretion in FHs 74 Int cells.

## Conclusions

Our findings reveal the role of P2XR in the induction of NLRP3 inflammasome activation in *T. gondii*-induced small intestinal epithelial cells. Furthermore, we found that the P2X7R/NLRP3 pathway plays an important role in IL-1β secretion and in the inhibition of *T. gondii* proliferation in infected cells (Fig. 6). These results may not only contribute to our understanding of the mucosal immune mechanism of *T. gondii* infection but also offer new insight into the identification of innate resistance in the gut epithelium.

## Additional files

Additional file 1: Table S1. Sequences of the primers used in this study. (DOC 32 kb)

Additional file 2: Figure S1. FHs 74 Int cells infected with *T. gondii* at higher infectious doses (MOI 20) reduced cell viability. FHs 74 Int cells were infected with the *T. gondii* GFP-RH strain at the indicated MOIs for 8 h, and the viability of FHs 74 Int cells were evaluated by an MTT assay. **Figure S2.** *Toxoplasma gondii*-induced IL-1β production in FHs 74 Int cells. FHs 74 Int cells were infected with *T. gondii* at an MOI of 10 for different time periods, and pro-IL-1β protein levels in the cell extracts were determined by Western blotting. **a** Representative blots presenting the protein levels of pro-IL-1β in mock-infected or *T. gondii*-infected FHs 74 Int cells (α-Tubulin served as an internal control for protein loading). **b** Bar plot depicting the pro-IL-1β/α-Tubulin ratio as determined by densitometric analysis of Western blotting and expressed as fold change compared with the mock-infection control. For all panels, data are presented as the mean ± SD. *P < 0.05, **P < 0.01, ***P < 0.001 compared with the uninfected control. All data are representative of three independent experiments. (ZIP 1710 kb)

## Abbreviations

ATCC: American Type Culture Collection
DMEM: Dulbecco’s modified Eagle’s medium
DMEM/F12: Dulbecco’s modified Eagle’s medium and nutrient mixture F12
ELISA: Enzyme-linked immunosorbent assay
FBS: Fetal bovine serum
FHs 74 Int cells: Non-transformed human fetal small intestinal epithelial cell line
HPRT1: Hypoxanthine phosphoribosyltransferase 1
HRP: Horseradish peroxidase
IL-1β: Interleukin-1β
MOI: Multiplicity of infection
MTT: 3-(4,5-dimethylthiazol-2-yl)-2,5-diphenyltetrazolium bromide
PBS: Phosphate-buffered saline
PVDF: Polyvinylidene fluoride
SDS-PAGE: Sodium dodecyl sulfate polyacrylamide gel electrophoresis
TBS: Tris-buffered saline
