# Supplementary material for: P2X7 receptor mediates NLRP3-dependent IL-1β secretion and parasite proliferation in Toxoplasma gondii-infected human small intestinal epithelial cells
Source: Parasit Vectors. 2018 Jan 2;11:1. doi: 10.1186/s13071-017-2573-y (PMC5748956; doi:10.1186/s13071-017-2573-y)
Supplement: Supplementary file 1 — Sequences of the primers used in this study. (DOC 32 kb) [file 13071_2017_2573_MOESM1_ESM.doc]

**Table S1.** Primers sequences used for real-timequantitative reverse transcriptase polymerase chain reaction (qRT-PCR)

| Gene  name | GeneBank Accession No. | Primer sequnence  (5’-3’) | Product size (bp) |
| --- | --- | --- | --- |
| NLRP1 | NM_014922.4 | F-ATACGAAGCCTTTGGGGACT  R-ACAAAGCAGAGACCCGTGTT | 148 |
| NLRP3 | XM_011544055.2 | F-AAAGGAAGTGGACTGCGAGA  R-TTCAAACGACTCCCTGGAAC | 129 |
| NLRC4 | XM_017004619.1 | F-GGAAAGTGCAAGGCTCTGAC  R-TGTCTGCTTCCTGATTGTGC | 129 |
| AIM2 | XM_005245616.4 | F-AGCCTGAACAGAAACAGATGG  R-CTTCTTGGGTCTCAAACGTGA | 120 |
| HPRT1 | NM_000194.2 | F-GACCAGTCAACAGGGGACAT  R-CTGCATTGTTTTGCCAGTGT | 111 |
