# Supplementary figures and images for: P2X7 receptor mediates NLRP3-dependent IL-1β secretion and parasite proliferation in Toxoplasma gondii-infected human small intestinal epithelial cells
Source: Parasit Vectors. 2018 Jan 2;11:1. doi: 10.1186/s13071-017-2573-y (PMC5748956; doi:10.1186/s13071-017-2573-y)

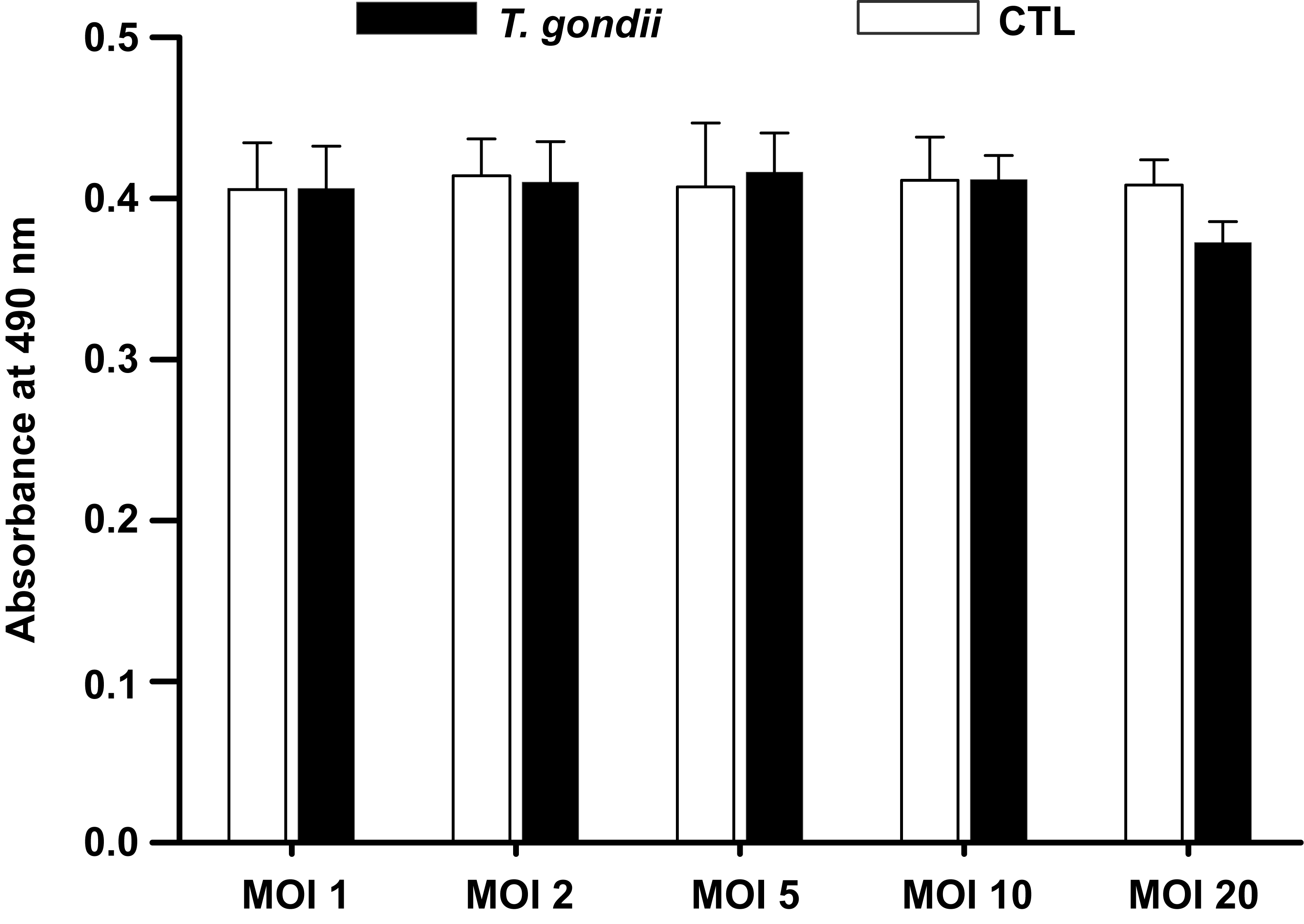

Supplement: Supplementary file 2 — FHs 74 Int cells infected with T. gondii at higher infectious doses (MOI 20) reduced cell viability. FHs 74 Int cells were infected with the T. gondii GFP-RH strain at the indicated MOIs for 8 h, and the viability of FHs 74 Int cells were evaluated by an MTT assay. Figure S2. Toxoplasma gondii-induced IL-1β production in FHs 74 Int cells. FHs 74 Int cells were infected with T. gondii at an MOI of 10 for different time periods, and pro-IL-1β protein levels in the cell extracts were determined by Western blotting. a Representative blots presenting the protein levels of pro-IL-1β in mock-infected or T. gondii-infected FHs 74 Int cells (α-Tubulin served as an internal control for protein loading). b Bar plot depicting the pro-IL-1β/α-Tubulin ratio as determined by densitometric analysis of Western blotting and expressed as fold change compared with the mock-infection control. For all panels, data are presented as the mean ± SD. *P < 0.05, **P < 0.01, ***P < 0.001 compared with the uninfected control. All data are representative of three independent experiments. (ZIP 1710 kb) [file 13071_2017_2573_MOESM2_ESM.zip › Fig S1 and S2/Fig S1.tif]

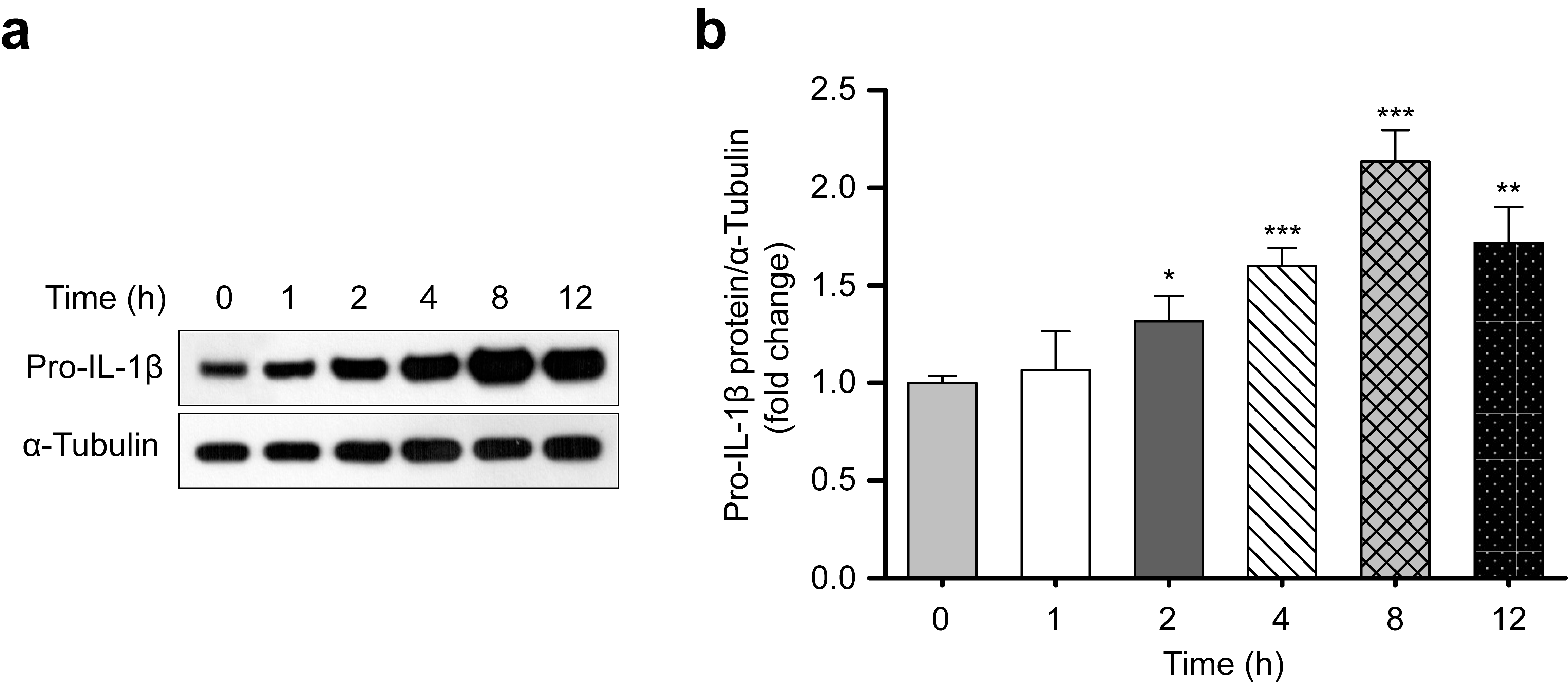

Supplement: Supplementary file 2 — FHs 74 Int cells infected with T. gondii at higher infectious doses (MOI 20) reduced cell viability. FHs 74 Int cells were infected with the T. gondii GFP-RH strain at the indicated MOIs for 8 h, and the viability of FHs 74 Int cells were evaluated by an MTT assay. Figure S2. Toxoplasma gondii-induced IL-1β production in FHs 74 Int cells. FHs 74 Int cells were infected with T. gondii at an MOI of 10 for different time periods, and pro-IL-1β protein levels in the cell extracts were determined by Western blotting. a Representative blots presenting the protein levels of pro-IL-1β in mock-infected or T. gondii-infected FHs 74 Int cells (α-Tubulin served as an internal control for protein loading). b Bar plot depicting the pro-IL-1β/α-Tubulin ratio as determined by densitometric analysis of Western blotting and expressed as fold change compared with the mock-infection control. For all panels, data are presented as the mean ± SD. *P < 0.05, **P < 0.01, ***P < 0.001 compared with the uninfected control. All data are representative of three independent experiments. (ZIP 1710 kb) [file 13071_2017_2573_MOESM2_ESM.zip › Fig S1 and S2/Fig S2.tif]
